# A Comparison of Psychiatric Comorbid Symptomology Between Adolescents With Restrictive/Avoidant Food Intake Disorder, Anorexia Nervosa and Atypical Anorexia Nervosa

**DOI:** 10.1002/erv.70014

**Published:** 2025-07-19

**Authors:** Daniel Wilson, Govind Krishnamorthy, Renata A. Mendes, Tania Withington, Melanie Dalton, Natalie J. Loxton

**Affiliations:** ^1^ Children's Health Queensland Hospital and Health Service Child and Youth Mental Health Service Eating Disorders Team Greenslopes Australia; ^2^ Child Health Research Centre University of Queensland South Brisbane Australia; ^3^ School of Applied Psychology Griffith University Brisbane Australia; ^4^ School of Psychology and Wellbeing University of Southern Queensland Ipswich Australia; ^5^ Centre for Health Research Manna Institute University of Southern Queensland Ipswich Australia

**Keywords:** adolescent, ARFID, atypical anorexia nervosa, child, eating disorder

## Abstract

**Objective:**

Psychiatric comorbid conditions are common among individuals with Eating Disorders (EDs), and these symptoms may exacerbate and/or interact with ED symptoms and impact treatment effectiveness. Whilst comorbid symptomology in Anorexia Nervosa (AN) has been well described, less is known about how the ‘newer’ ED diagnoses of Atypical Anorexia Nervosa (AAN) and Avoidant/Restrictive Food Intake Disorder (ARFID) differ relative to AN. The current study aimed to extend the literature by examining similarities and differences in comorbid symptomology AN, AAN, and ARFID groups.

**Method:**

In this cross‐sectional study, young people (*n* = 311, female = 87.8%, mean age = 14.32: SD = 2.05, range 5–17) with AN, AAN, and ARFID completed self‐report measures capturing comorbid symptomology prior to engaging in treatment at a specialist ED outpatient clinic.

**Results:**

There was no difference between AN and AAN on any measure of comorbid symptoms. Both showed severe levels of comorbidity with over half exceeding the cut‐point for four or more comorbid diagnoses, with Obsessive Compulsive Disorder, depression and Social Anxiety particularly common. ARFID, on the other hand, had comparatively less severe comorbid symptoms compared to AN and AAN.

**Conclusions:**

Findings provide a better understanding of the nature of comorbid symptomology among these disorders and encourages future research to investigate the role that they play in the treatment outcome.

## Summary

- Young people (*n* = 311) with AN, AAN, and ARFID completed self‐report measures capturing comorbid symptomology prior to engaging in treatment at a specialist ED outpatient clinic
- AN and AAN did not differ on any measure of comorbid symptoms.
- AN and AAN showed severe levels of comorbidity with over half exceeding the cut‐point for four or more comorbid diagnoses with Obsessive Compulsive Disorder, depression and Social Anxiety particularly common.
- Young people with ARFID had comparatively less severe comorbid symptoms compared to AN and AAN.

## 1 Introduction

Anorexia nervosa (AN), atypical anorexia nervosa (AAN) and avoidant and restrictive food intake disorder (ARFID) are distinct disorders according to the Diagnostic and Statistical Manual of Mental Disorders (DSM‐5‐TR: A. P. Association 2022). Whilst they can share common symptomology of weight loss, restriction and/or avoidance of dietary intake, in AAN and AN, this is commonly motivated by weight and/or shape concerns, whereas in ARFID, dietary abnormalities are typically motivated by sensory aversion, avoidance of feared consequences, and/or lack of hunger/food interest (A. P. Association 2022). Individuals with AN and AAN typically experience extreme weight and shape concerns and can experience weight loss due to restrictive eating or weight control behaviours; however, in AAN, the individual's weight remains in or above the normal range despite significant weight loss (A. P. Association 2022).

Understanding psychiatric comorbid conditions and symptoms of individuals with these disorders is important, as symptoms may exacerbate and/or interact with ED symptoms and impact treatment effectiveness and/or completion (Day et al. 2024). For example, young people with comorbid psychiatric conditions require a longer course of Family Based Treatment (FBT) compared to those who do not have comorbid conditions (Lim et al. 2023), and those with external comorbid psychopathology interfering with treatment require a longer course of enhanced Cognitive Behaviour Therapy (CBT‐E; Dalle Grave and Calugi 2020).

A recent rapid review of the literature by Hambleton et al. (2022) investigated medical and psychiatric comorbidities of EDs. Results showed that across ED subtypes, the most common comorbid psychiatric conditions were anxiety, mood, post‐traumatic stress, and substance use disorders. This review demonstrated that whilst there is extensive literature investigating comorbidities in AN, there is a significant gap in our understanding and research into comorbidities within less studied diagnostic subgroups like AAN and ARFID. By determining similarities and differences between these groups, we can tailor treatment approaches to address the unique and common needs of each subgroup. This is particularly crucial as current treatments for the relatively novel diagnoses of ARFID and AAN currently mirror those used for AN, such as FBT and CBT‐E. A better understanding of how these groups compare to each other and AN may inform a need for targeted treatment modifications that better cater to the unique aspects of each disorder.

## 2 Comparisons of Comorbid Symptomology Between AN, AAN and ARFID

Most of the research comparing comorbid symptomology among these subtypes has focussed on contrasting AN with AAN. On global ED psychopathology as measured by either the Eating Disorders Examination (EDE: Fairburn and Beglin 1994) or Eating Disorders Inventory‐3 (EDI‐3: Garner 1 991), results are mixed with some studies finding no differences between adolescent groups on global EDE scores (e.g., Keery et al. 2019) and some studies finding higher scores for those with AAN compared to AN (e.g., EDE‐Global: Sawyer et al. 2016; EDI‐3: Zanna et al. 2021). Similar mixed results are found in adult populations with a general trend toward AAN groups reporting greater ED symptom severity than AN groups (Walsh et al. 2023).

With regards to symptoms of psychiatric co‐morbidity, data suggest that there are few differences between AN and AAN, with no differences reported in adolescent populations on measures of depressive, anxiety, and obsessive‐compulsive symptomology (Billman Miller et al. 2024; Sawyer et al. 2016; Walsh et al. 2023). There are some discrepant results between adolescent and adult populations, with some adult studies reporting higher levels of Obsessive‐Compulsive Disorder (OCD) symptoms in AN populations compared to AAN (Fitterman‐Harris et al. 2024), whereas other studies found no differences in adolescent populations (e.g., Sawyer et al. 2016). However, there are insufficient studies to draw strong conclusions as to adult versus adolescent differences. Beyond anxiety, depression, and OCD symptoms, few studies have compared groups on measures of other comorbid conditions with high prevalence in ED (e.g., post‐traumatic stress disorder [PTSD], personality disorders; Hambleton et al. 2022).

When looking at comparisons with individuals with ARFID, most of the existing literature has focussed on differences in comorbid symptoms between AN and ARFID. Results indicate that individuals with AN report higher symptoms of both depression and anxiety compared to ARFID. However, it appears that those with ARFID are more likely to be diagnosed with an anxiety disorder, Autism Spectrum Disorder (ASD) or Attention‐Deficit Hyperactivity Disorder (ADHD), whereas those with AN are more likely to be diagnosed with a mood disorder (e.g., Becker et al. 2019; Bryson et al. 2018; Cañas et al. 2021; Lieberman et al. 2019).

Few studies have evaluated comorbid symptomology and diagnoses among the three groups. Keery et al. (2019) compared adolescents with ARFID, AAN, and AN on measures including the EDE‐Q, depression (Children's Depression Inventory; Kovacs 2015) and anxiety (Beck Anxiety Inventory; Beck et al. 1988). AAN and AN groups were combined to preserve sample size, given they found no comorbid symptom differences between the two groups. They found that the ARFID group scored lower on symptoms of depression, anxiety, and perfectionism, and higher on self‐esteem measures compared to the combined AN/AAN group. The ARFID group were also less likely to have a diagnosis of a depressive disorder, and more likely to have an ADHD diagnosis. However, they found no difference between groups on rates of anxiety or ASD diagnoses, which differed from other results finding increased prevalence of ASD in ARFID relative to AN (e.g., Becker et al., 2019; Bryson et al. 2018; Cañas et al. 2021; Lieberman et al. 2019). Keery and colleagues highlighted the need for further research to replicate and expand their findings given the lack of existing research in this area.

Zanna et al. (2021) compared ED specific and comorbid symptoms between ARFID, AAN and AN in a sample of adolescent patients from a tertiary care ED programme in Italy. They found that the ARFID group was younger and had fewer depressive symptoms relative to AN and AAN and had more ADHD symptoms and fewer OCD symptoms relative to the AN group but not the AAN group. While there were no differences across the groups on overall anxiety symptoms, the ARFID group scored higher than the AN group on separation/panic symptoms. Higher scores were found on the EDI subscales of Drive for Thinness, Bulimia, and Body Dissatisfaction in the AAN group, followed by the AN group, with the ARFID group scoring lowest. There were no differences between the three groups on EDI subscales of the Emotional Dysregulation, Maturity Fears, Interpersonal Insecurity, and Interoceptive Deficits. The ARFID group was also more likely to have a diagnosed anxiety disorder than the AN group, but not compared to the AAN group. There was no difference in the rate of mood disorders between groups. The authors suggested these findings may indicate that there may be psychopathology common across the continuum of restrictive EDs characterised by negative affectivity and internalisation, which if confirmed could have implications for early and modular treatment targets. The authors suggested further research to see if these findings generalise into outpatient settings.

Additional investigation of the comorbid symptomology of ED populations is required to further understand the temporal nature, shared psychopathology, and interaction between EDs and comorbid symptoms. In Australian settings, there is no study that compares comorbid symptomology among AN, AAN, and ARFID groups. More broadly, few studies have reported on other comorbid symptomology or diagnoses commonly associated with EDs (e.g., PTSD, Borderline Personality Disorder [BPD]), and so little is known about the similarities or differences among AN, AAN, and ARFID in these realms. To date, treatment of AAN is almost entirely based on treatment of AN (e.g., Dalle Grave et al. 2015; Hughes et al. 2017). Similarly, treatments for ARFID among children and adolescents are only recently emerging, and in part have been adapted from, and informed by, existing treatments for AN (e.g., FBT; Van Wye et al. 2023). It is important to understand the similarities and differences among these disorders to help inform the degree to which treatments need to be altered according to group, and to what degree the commonalities between these groups may be well targeted by existing treatments.

The current study aimed to extend the literature by describing and comparing the comorbid symptomology among AN, AAN, and ARFID groups using a comprehensive range of measures in an adolescent Australian cohort presenting for treatment at a specialist tertiary outpatient clinic. It was hypothesised that the AN and AAN groups would score higher on measures of depression, anxiety, and OCD compared to the ARFID group. Further, it was expected that all groups would differ on measures of ED psychopathology, with the AAN group scoring higher than the AN group, who in turn were expected to score higher than the ARFID group. Additionally, a broader range of measures for comorbid conditions and constructs relevant to either treatment outcome (e.g., expressed emotion and food related obsessions and compulsions as in FBT) or treatment processes (e.g., interpersonal difficulties, perfectionism, low self‐esteem, mood intolerance as in CBT‐E broad form) were included and tested for exploratory purposes. As there has been little use of these measures in similar studies, no specific hypotheses were made regarding these analyses.

## 3 Methods

### 3.1 Participants and Procedure

The sample consisted of 311 participants (female = 87.8%, mean age = 14.32: SD = 2.05, range 5–17), who were consecutive referrals to a public outpatient child and youth specialist ED service. Participants referred to the clinic were assessed by a multidisciplinary team with experience in assessing and treating child and adolescent EDs. Assessment included diagnostic evaluation with the young person and family, anthropometric and medical evaluation, as well as completion of self‐report measures. Further details about the clinic procedure are described in Lim et al. 2023. Only those with a diagnosis of AN (*n* = 88), AAN (*n* = 188) and ARFID (*n* = 35) and who consented to research were included for the current study. The International Classification of Diseases and Related Health Problems (10th ed.; ICD‐10; World Health Organization 2016) is utilised in the clinic, and as such these criteria were strictly applied for AN and AAN diagnoses. A weight cut‐off for diagnosing AN was established at less than fifth centile BMI. The ICD also states that AN should be diagnosed if a person's weight is 15% below that expected (either lost or failed to make expected weight gain through developmental period). This is consistent with the DSM‐5‐TR guidelines which state individuals above the fifth centile may be judged to be significantly underweight (and thus meet criteria for AN diagnosis) if they have failed to maintain expected growth trajectory (as long as BMI remains below median BMI centile for age). As such, AN was also diagnosed if the young person's weight was 15% below that expected (and their BMI was below the 50th centile for age and gender), with expected body weight calculated using Centre for Disease Control and Prevention growth charts (www.cdc.gov/growthcharts) if premorbid weight data were available through previous medical records. AAN diagnosis was also applied as per ICD‐10 criteria (i.e., core features of AN present, but with weight above fifth centile BMI and no evidence of failure to meet expected growth rate by more than 15%). The DSM 5 criteria were utilised to diagnose ARFID given that there are no criteria under ICD‐10.

## 4 Measures

*Demographic and Clinical Variables*. Participants responded to items relating to their age and sex. Clinical data were collected from the assessing clinician, which included psychiatric diagnoses and weight/height history.

*Body Mass Index* (*BMI*) *centiles*. BMI centiles were calculated using an individual's weight, height, and the Centre for Disease Control and Prevention growth charts (www.cdc.gov/growthcharts).

*Eating Disorders Examination Questionnaire* (*EDE‐Q*; *Fairburn and Beglin* 1994; *Carter et al.* 2001). The EDE‐Q used in this study is the adolescent version adapted by Carter et al. (2001), which is a modification of the original adult version by Fairburn and Beglin (1994). This adolescent version shortens the symptom reporting period from 28 to 14 days and simplifies the language of some items to better suit younger respondents. The EDE‐Q is a 36‐item questionnaire comprising four subscales: Restraint, Eating Concern, Shape Concern, and Weight Concern. It is scored on a 7‐point Likert scale (0–6), with higher scores indicating greater severity of ED symptoms. Mean scores are used for each subscale and for the global score. The internal reliability for the global score was 0.97.

*Clinical Impairment Assessment* (*CIA*). The CIA is a 16‐item self‐report measure of the degree of psychosocial impairment due to ED features (Bohn et al. 2008). Items are scored on a 4‐point Likert scale (0–3), and are summed to form a total score, with higher scores equating to higher levels of impairment. The internal reliability was 0.96.

*Patient Health Questionnaire 9* (*PHQ‐9*). The PHQ‐9 is a 9‐item self‐report measure and was used to measure symptoms of depression (Kroenke et al. 2001). Items are scored on a 4‐point Likert scale (0–3) and are summed for a total score, with higher scores indicating greater depressive symptoms. The recommended clinical cut point of 10 is utilised in this study as indicative of clinically significant symptoms. The internal reliability was 0.88.

*Child Anxiety Scale* (*CAS‐8*). The CAS‐8 is an 8‐item self‐report measure of anxiety (Reardon et al. 2018). It is scored on a 4‐point Likert scale with higher scores indicating greater anxiety symptoms. The internal reliability was 0.96.

*Social Anxiety* (*SCAS*). The social anxiety subscale from the Spence Children's Anxiety Scale (SCAS; Spence et al. 2003) was used to measure social anxiety. The scale consists of five items scored on a 4‐point scale, with higher scores reflective of higher social anxiety. The internal reliability was 0.81.

*Children's PTSD Symptoms Scale for DSM‐5* (*CPSS‐SR‐5*). The CPSS‐SR‐5 is a 20‐item self‐report measure of PTSD symptoms (Foa et al. 2018). Scores are summed to form a global score, with authors recommending that a cut‐point of above 31 be used to distinguish clinically significant symptoms. The internal reliability was 0.96. As opposed to the other measures in the study, young people only responded to the items in the CPSS if they indicated that they had experienced a traumatic event, which is operationalised with details and examples in the questionnaire. 48.4% of young people provided responses to this questionnaire.

*Obsessive Compulsive Inventory–Child Version* (*OCI‐CV‐R*). The OCI‐CV‐R is an 18‐item self‐report measure of OCD symptoms (Abramovitch et al. 2022; Foa et al. 2010). Scores are summed to form a global score, with authors recommending that a cut‐point of above 8 be used to distinguish those with an OCD diagnosis from clinical controls. The internal reliability was 0.93.

*Borderline Personality Features Scale for Children‐11* (*BPFSC‐11*). The BPFSC‐11 is an 11‐item self‐report measure of BPD symptoms (Sharp et al. 2014). Scores are summed to form a global score, with authors recommending that a cut‐point of above 34 be used to distinguish clinically significant symptoms. The internal reliability was 0.92.

*CAGE‐Adapted to Include Drugs* (*CAGE*). The CAGE (Brown and Rounds 1995) is a four item yes/no response scale, with items related to substance use/dependence. The internal reliability was 0.83.

*Strengths and Difficulties Questionnaire–Hyperactivity/inattention subscale* (*SDQ‐HI*). The SDQ‐HI is a 5‐item self‐report measure of hyperactive and inattentive symptoms consistent with ADHD (Goodman et al. 2003). Scores are summed to form a global score, with authors recommending that a cut‐point of above 7 be used to distinguish clinically significant symptoms when using the self‐report version. The internal reliability was 0.80.

*Yale‐Brown‐Cornell Eating Disorder Scale–Self‐Report* (*YBC‐EDS‐SR*). The YBC‐EDS‐SR (Fitzpatrick and Weltzin 2014) is a 17‐item self‐report measure of obsessions and compulsions related to eating, which has previously been found to be a moderator of treatment outcome (Le Grange et al. 2012). Items used for this study were the 8 items that comprise the global eating obsession and rituals subscale, which demonstrated excellent internal reliability (*α* = 0.92).

*Brief Dyadic Scale of Expressed Emotion* (*BDSEE*). The BDSEE measures the young person's perceptions of their maternal caregivers expressed emotions. The subscale used measured perceived Warmth (*α* = 0.89), Overinvolvement (*α* = 0.83), and Criticism (*α* = 0.77). Higher scores equates to greater perceived level of the domain (i.e., higher score = greater perceived criticism; Medina‐Pradas et al. 2011).

*Frost Multidimensional Perfectionism Scale* (*FMPS*). The Concerns over Mistakes (9 items: *α* = 0.95); and Personal Standards (7 items: *α* = 0.89) subscales were used from the FMPS (Frost et al. 1993). Items are summed with higher scores indicating higher perfectionism.

*Rosenburg Self‐Esteem Scale* (*RSES*). The RSES (Rosenberg 1986) is a 10‐item self‐report scale measuring self‐esteem. Items are summed with higher scores indicating higher self‐esteem. The internal reliability was 0.87.

*Compulsive Exercise Test* (*CET*). The CET (Taranis et al. 2011) is a 24‐item measure of driven/obsessive exercise comprised of five subscales (Avoidance and Rule‐Driven behaviours, Weight control, Mood Improvement, Lack of Exercise Enjoyment, and Rigidity) and a global score. Items are averaged to form subscale scores, which are summed for a global score. The internal reliability was 0.95.

*Distress Intolerance Inventory–Youth* (*DII‐Y*). The DII‐Y (Keller et al. 2019) is a 10‐item measure capturing difficulties tolerating strong emotions. Items are summed to form a total score, with higher scores indicating more difficulties with distress tolerance. The internal reliability was 0.94.

*Paediatric Quality of Life Inventory Short Form 15–Young Person* (*PedsQL‐SF15*). The PedsQL‐SF15 (Varni et al. 2001) is a 15‐item measure comprising four subscales representing domains of quality of life: physical, emotional, social, and school functioning. Items are summed to form the subscales and a total score, with higher scores indicating greater impairment to quality of life. The internal reliability was 0.89.

*Detail Flexibility Questionnaire* (*DFlex*). The DFlex (Roberts et al. 2011) is a 24‐item measure representing two domains of neurocognitive functioning that are captured by two subscales: cognitive rigidity (*α* = 0.89) and attention to detail (*α* = 0.91).

*Interpersonal Relationships in Eating Disorders* (*IR‐ED*). The IR‐ED (Jones et al. 2019), is a 15‐item self‐report measure assessing interpersonal difficulties in the context of an ED. Items are averaged to form a global score, with higher scores representing greater difficulties (*α* = 0.93).

### 4.1 Analytic Plan

Data were analysed using SPSS version 30. While several outliers were detected, they were maintained in the analysis, as they did not change the interpretation of the results. Assumptions of normality and homogeneity of variance were assessed using normal Q‐Q plots and Box's test of equality of covariance matrices (*p* = 0.063), respectively; no violations were detected. There were no instances of multicollinearity (greater than 0.9), and no multivariate outliers were identified by Mahalanobis distance (*p* > 0.001). Analysis of variance (ANOVA) was utilised to assess for differences between groups on continuous variables, with *F* tests interpreted with a Bonferroni adjusted *p* value > 0.002 (0.05/27 = 0.0019). Age was included as a covariate as previous research has found that group comorbidity differences between ARFID and AN may be explained by age (Kambanis et al. 2022). Significant interactions were followed up with a post hoc test with Bonferroni adjustments applied for multiple comparisons (*p* values adjusted by SPSS and interpreted as significant at less than *p* = 0.05). Chi‐square was used to test for group differences on categorical variables.

### 4.2 Ethics

The project was approved by the Children's Health Queensland Hospital and Health Service Human Research Ethics Committee (HREC/20/QCHQ/67708). Informed consent was obtained from parents/legal guardians as per ethical approval.

## 5 Results

### 5.1 Demographic, Physical and Eating Disorder Related Variables

As shown in Table 1, the ARFID group was younger and had a higher proportion of males than the AAN and AN groups, who did not differ from one another (NOTE: The main analyses were run with and without age and sex as covariates with no difference to the interpretation of the results). BMI centile was higher in those with AAN compared to both the AN and ARFID groups, who did not differ from one another. The ARFID group scored lower on the EDE‐Q and CIA compared to the AN and AAN groups, who did not differ from one another.

**TABLE 1:** Means, standard deviations and group differences on demographic and ED related variables.

Measure	AN			AAN		ARFID		ANOVA/Chi square	Partial eta squared	Post‐hoc comparisons (Bonferroni adjusted *p* value)
	N		M (SD)	N	M (SD)	N	M (SD)			
Age	88		14.75 (1.64)	188	14.56 (1.55)	35	11.86 (3.34)	F(2,308) = 34.82, *p* < 0.001*	**0.18**	**AN versus ARFID, *p* < 0.001**
										**AAN versus ARFID, *p* < 0.001**
Sex (% female)			93.2%		90.4%		60.0%	Chi square (2) = 28.80, *p* < 0.001*		**AN versus ARFID, *p* < 0.05**
										**AAN versus ARFID, *p* < 0.05**
BMI centile	88		13.32 (13.56)	188	52.37 (24.85)	35	22.25 (27.17)	F(2,307) = 99.56, *p* < 0.001*	**0.39**	**AAN versus ARFID, *p* < 0.001**
										**AAN versus AN, *p* < 0.001**
EDE‐Q global	85		3.42 (1.73)	184	3.72 (1.67)	28	0.49 (0.64)	F(2,293) = 40.50, *p* < 0.001*	**0.22**	**AN versus ARFID, *p* < 0.001**
										**AAN versus ARFID, *p* < 0.001**
Clinical impairment assessment	84		28.92 (13.28)	183	29.36 (12.98)	28	8.71 (8.67)	F(2,291) = 27.12, *p* < 0.001*	**0.16**	**AN versus ARFID, *p* < 0.001**
										**AAN versus ARFID, *p* < 0.001**

*Note:* After Bonferroni correction, *F* tests were only followed up if significant at *p* < 0.002 which is denoted with * and bold text. Post‐hoc comparisons show Bonferroni adjusted *p* values and are interpreted as significant at *p* < 0.05.

Abbreviations: AAN = atypical anorexia nervosa, AN = anorexia nervosa, ANOVA = analysis of variance, ARFID = avoidant/restrictive food intake disorder, BMI = body mass index, EDE = eating disorders examination.

### 5.2 Symptomology Related to DSM‐5 Diagnoses

As shown in Table 2, there were no difference between AAN and AN groups on any of the measures capturing symptoms of comorbid DSM5 diagnoses. The ARFID group scored lower than both the AAN and AN groups on measures of depression, generalised anxiety, social anxiety, PTSD, OCD and BPD. There were no group differences on measures of ADHD or substance use. The proportions of participants above the clinical cut‐points for these measures are shown in Table 3 and the proportions for overall number of comorbid thresholds exceeded are shown in Table 4. Just over half of both the AN and AAN groups exceeded the cut‐point for four or more comorbid disorders with OCD the most common cut‐point exceeded, whereas with ARFID, roughly two thirds exceeded only one or no comorbid cut‐points, with OCD again the most common.

**TABLE 2:** Means, standard deviations and group differences on measures related to DSM5 diagnoses.

Measure	AN		AAN		ARFID		ANOVA	Partial eta squared	Post‐hoc comparisons (Bonferroni adjusted *p* value)
	N	M (SD)	N	M (SD)	N	M (SD)			
PHQ9 (depression)	84	13.43 (7.06)	182	15.24 (7.01)	28	6.11 (4.97)	*F*(2,290) = 16.18, *p* < 0.001*	**0.10**	**AN versus ARFID, *p* < 0.001**
									**AAN versus ARFID, *p* < 0.001**
CAS (generalised anxiety)	83	13.86 (6.03)	176	14.14 (6.13)	27	8.07 (5.25)	*F*(2,282) = 9.32, *p* = < 0.001*	**0.06**	**AN versus ARFID, *p* < 0.001**
									**AAN versus ARFID, *p* < 0.001**
SCAS (social anxiety subscale)	83	10.37 (4.96)	176	11.40 (4.66)	27	6.67 (4.42)	*F*(2,282) = 21.98, *p* < 0.001*	**0.06**	**AN versus ARFID, *p* = 0.02**
									**AAN versus ARFID, *p*** < **0.001**
OCI‐ CV (OCD)	88	13.52 (7.39)	187	14.45 (9.52)	28	7.78 (5.96)	*F*(2,299) = 6.86, *p* < 0.001*	**0.04**	**AN versus ARFID, *p*** < **0.01**
									**AAN versus ARFID, *p* < 0.001**
CPSS SR‐5 (PTSD) % responded		44.3%		50.5%		46.7%	Chi square (2) = 0.96, *p* = 0.62		—
CPSS SR‐5 (PTSD)	43	33.84 (24.31)	102	37.42 (22.64)	18	11.94 (11.06)	*F*(2,159) = 7.45, *p* < 0.001*	**0.08**	**AN versus ARFID, *p* = 0.02**
									**AAN versus ARFID, *p* < 0.001**
BPFSC‐11 (BPD)	84	31.99 (10.89)	178	33.48 (10.86)	27	19.89 (7.85)	*F*(2,285) = 15.78, *p* < 0.001*	**0.10**	**AN versus ARFID, *p* < 0.001**
									**AAN versus ARFID, *p*** **<** **0.001**
SDQ inattention/hyperactivity (ADHD)	77	6.06 (3.24)	158	7.25 (3.22)	24	6.45 (2.70)	*F*(2,255) = 4.08, *p* = 0.02	0.03	—
CAGE (substance use)	78	0.33 (0.96)	161	0.42 (0.99)	25	0.04 (0.20)	*F*(2,260) = 0.76, *p* = 0.46	0.01	—

*Note:* After Bonferroni correction, *F* tests were only followed up if significant at *p* < 0.002 which is denoted with * and bold text. Post‐hoc comparisons show Bonferroni adjusted *p* values and are interpreted as significant at *p* < 0.05.

Abbreviations: AAN = atypical anorexia nervosa, AN = anorexia nervosa, ANOVA = analysis of variance, ARFID = avoidant/restrictive food intake disorder, BPFSC‐11 = borderline personality features scale–child version 11, SDQ = strengths and difficulties questionnaire, CAS8 = children's anxiety scale–8, CPSS SR‐5 = child post‐traumatic stress scale self‐report, OCI‐CV = obsessive compulsive inventory–child version, PHQ9 = patient health questionnaire −9, SCAS = social anxiety subscale.

**TABLE 3:** Proportion of young people above clinical cut‐point for comorbidity measures.

Measure	AN	AAN	ARFID
PHQ9 (depression)	61.4%	69.7%	17.1%
CAS (generalised anxiety)	40.9%	42.6%	8.6%
SCAS (social anxiety)	55.7%	62.8%	17.1%
OCI‐ CV (OCD)	78.4%	70.2%	42.9%
CPSS SR‐5 (PTSD)	27.4%	34.0%	4.4%
BPFSC‐11 (BPD)	39.8%	50.0%	5.7%
SDQ inattention/hyperactivity (ADHD)	44.3%	53.7%	28.6%

Abbreviations: AAN = atypical anorexia nervosa, AN = anorexia nervosa, ARFID = avoidant/restrictive food intake disorder, BPFSC‐11 = borderline personality features scale–child version 11, CAS8 = children's anxiety scale–8, CPSS SR‐5 = child post‐traumatic stress scale self‐report, OCI‐CV = obsessive compulsive inventory–child version, PHQ9 = patient health questionnaire −9, SCAS = social anxiety subscale, SDQ = strengths and difficulties questionnaire.

**TABLE 4:** Number of clinical cut‐points exceeded for measures related to comorbid conditions.

Number of cut‐points exceeded	AN		AAN		ARFID	
	%	Cumulative %	%	Cumulative %	%	Cumulative %
0/7	10.2	10.2	10.6	10	40.0	40.0
1/7	18.2	28.4	7.4	18.1	25.7	65.7
2/7	11.4	39.8	11.2	29.3	22.9	88.6
3/7	9.1	48.8	11.7	41.0	2.9	91.4
4/7	14.8	63.6	15.4	56.4	2.9	94.3
5/7	12.5	76.1	16.5	72.9	2.9	97.1
6/7	11.4	87.5	17.0	89.9	2.9	100
7/7	12.5	100	10.1	100	0	100

Abbreviations: AAN = atypical anorexia nervosa, AN = anorexia nervosa, ARFID = avoidant/restrictive food intake disorder.

### 5.3 Symptomology Related to Other Psychological Constructs

As shown in Table 5, there were no differences between AAN and AN groups on any of the measures. The ARFID group scored lower than the AN and AAN groups on measures of self‐esteem, interpersonal relationship difficulties, compulsive exercise, distress intolerance, both perfectionism subscales, and eating‐related obsessions and rituals. There were no difference between the groups on measures of expressed emotion, detail flexibility, or quality of life.

**TABLE 5:** Means, standard deviations and group differences on measures related to psychological/family constructs.

Measure	AN		AAN		ARFID		ANOVA	Partial eta squared	Post‐hoc comparisons (Bonferroni adjusted *p* value)
	N	M (SD)	N	M (SD)	N	M (SD)			
Distress intolerance inventory–youth	78	28.38 (10.45)	161	29.67 (10.45)	25	20.56 (9.58)	*F*(2,260) = 7.67, *p* < 0.001*	**0.06**	**AN versus ARFID, *p* < 0.01**
									**AAN versus ARFID, *p* < 0.001**
Frost multidimensional perfections scale (personal standards)	81	21.35 (7.28)	171	22.16 (6.48)	25	16.32 (6.02)	*F*(2,273) = 8.20, *p* < 0.001*	**0.06**	**AN versus ARFID, *p* < 0.01**
									**AAN versus ARFID, *p* < 0.001**
Frost multidimensional perfections scale (concerns over mistakes)	81	28.93 (10.47)	171	30.67 (8.99)	25	17.64 (8.71)	*F*(2,273) = 17.03, *p* < 0.001*	**0.11**	**AN versus ARFID, *p* < 0.001**
									**AAN versus ARFID, *p* < 0.001**
Brief dyadic scale of expressed emotion (perceived criticism)	83	18.07 (9.40)	178	20.23 (8.56)	27	14.33 (5.71)	*F*(2,284) = 4.76, *p* = 0.09	0.03	—
Brief dyadic scale of expressed emotion (perceived overinvolvement)	83	23.77 (11.74)	178	27.46 (13.54)	27	18.67 (10.70)	*F*(2,284) = 5.91, *p* = 0.004	0.04	
Brief dyadic scale of expressed emotion (perceived warmth)	83	31.93 (7.74)	178	31.51 (8.03)	27	33.85 (5.38)	*F*(2,284) = 0.44, *p* = 0.64	< 0.01	—
Detail flexibility questionnaire (cognitive rigidity)	83	45.19 (13.51)	175	43.91 (13.86)	25	35.48 (12.34)	*F*(2,279) = 4.71, *p* = 0.01	0.03	
Detail flexibility questionnaire (attention to detail)	83	43.23 (13.27)	175	42.04 (14.66)	25	33.92 (14.66)	*F*(2,279) = 3.77, *p* < 0.02	0.03	
YBC‐EDS‐SR (total obsessions and rituals)	60	16.28 (7.48)	140	15.23 (7.61)	22	5.55 (4.28)	*F*(2,218) = 15.03, *p* < 0.001*	**0.12**	**AN versus ARFID, <** **0.001**
									**AAN versus ARFID, *p* < 0.001**
YBC‐EDS‐SR (ego‐syntonic)	67	4.19 (2.39)	135	4.41 (2.36)	20	1.40 (1.67)	*F*(2,218) = 12.47, *p* < 0.001*	**0.10**	**AN versus ARFID, <** **0.001**
									**AAN versus ARFID, *p* < 0.001**
YBC‐EDS‐SR (motivation to change)	67	11.69 (5.41)	135	11.69 (5.86)	22	6.05 (5.04)	*F*(2,220) = 8.01, *p* < 0.001*	**0.07**	**AN versus ARFID, *p* < 0.001**
									**AAN versus ARFID, *p* < 0.001**
Rosenburg self‐esteem scale	80	21.82 (6.28)	166	21.63 (5.50)	25	29.40 (4.68)	*F*(2,267) = 15.44, *p* < 0.001*	**0.10**	**AN versus ARFID, *p* < 0.001**
									**AAN versus ARFID, *p* < 0.001**
Compulsive exercise scale	80	11.20 (5.44)	164	12.21 (5.16)	24	6.06 (3.08)	*F*(2,264) = 14.06, *p* < 0.001*	**0.10**	**AN versus ARFID, *p* < 0.001**
									**AAN versus ARFID, *p* < 0.001**
Interpersonal relationships in eating disorders	81	3.15 (1.10)	167	3.23 (1.08)	25	1.86 (0.63)	*F*(2,269) = 16.45, *p* < 0.001*	**0.11**	**AN versus ARFID, *p* < 0.001**
									**AAN versus ARFID, *p* < 0.001**
PEDSQL‐SF15	64	53.61 (20.82)	123	51.27 (16.89)	19	65.97 (16.66)	*F*(2, 202) = 3.72, *p* = 0.03	0.04	

*Note:* After Bonferroni correction, *F* tests were only followed up if significant at *p* < 0.002 which is denoted with * and bold text. Post‐hoc comparisons show Bonferroni adjusted *p* values and are interpreted as significant at *p* < 0.05.

Abbreviations: AAN = atypical anorexia nervosa, AN = anorexia nervosa, ANOVA = analysis of variance, ARFID = avoidant/restrictive food intake disorder, PEDSQL‐SF15 = paediatric quality of life inventory–short form, YBC‐EDS‐SR = Yale‐brown‐Cornell eating disorders scale–self report.

## 6 Discussion

The main aim of the study was to compare comorbid symptoms among AN, AAN, and ARFID groups on measures of comorbid symptomology related to diagnoses (e.g., depression, anxiety, PTSD, BPD), or psychological or family constructs of relevance to the treatment process or outcome in FBT (e.g., food related obsession and compulsions, expressed emotion) or CBT‐E (perfectionism, interpersonal difficulties, mood intolerance).

### 6.1 Similarities and Differences in Comorbid Symptomology

It was hypothesised that the AN and AAN groups would score higher on measures of depression, anxiety and OCD relative to the ARFID group. Results supported the hypotheses, with the ARFID group scoring lower on measures of depression, OCD and anxiety (social and generalised) compared to the AN and AAN groups. Further, the ARFID group scored less severely than the AAN and AN groups on nearly all of the reported constructs including symptoms of co‐morbid diagnoses (i.e., PTSD, BPD) as well as other psychological constructs (i.e., perfectionism, self‐esteem, compulsive exercise, and interpersonal difficulties). This may indicate a higher subjective level of distress and psychological impairment associated with AN and AAN compared to ARFID, which is supported by results on measures of clinical impairment.

There was no difference between groups on measures of ADHD, detail flexibility and expressed emotion. The results on symptoms of ADHD and detail flexibility may represent a common risk factor that predisposes young people to developing abnormal and restrictive eating patterns. Alternatively, the measure may be capturing some of the ‘starvation syndrome’ symptoms associated with restrictive eating patterns (Keys et al. 1950). Further research into the change of these symptoms across treatment with restoration to healthy weight and eating patterns may provide more information with respect to these interpretations. The similarities in expressed emotion indicate commonalities among families with EDs. This could mean that caring for a child with eating difficulties gives rise to similar difficulties with expressed emotion regardless of the diagnosis. Conversely, it could mean that difficulties with expressing emotion in the family environment represents a risk factor for developing future eating problems. Further longitudinal research could clarity temporal precedence.

Expressed emotion has been demonstrated to be a poor prognostic factor across FBT for AN, and treatment modification/adjuncts are recommended (Aarnio‐Peterson et al. 2024). The current results indicate similar levels of expressed emotion in ARFID as in AN/AAN, and may indicate that similar considerations may be warranted in family‐based treatment for ARFID; however, more research confirming the role of expressed emotion in ARFID treatment outcome is required.

### 6.2 Similarities and Differences in ED Symptomology

It was expected that the AAN group would score higher than the AN group, who in turn were expected to score higher than the ARFID group. Results partially supported the hypothesis related to ED psychopathology and clinical impairment, with the AN and AAN groups scoring higher than the ARFID groups, which was expected and consistent with previous results (Keery et al. 2019; Lieberman et al. 2019; Zanna et al. 2021). This highlights that restrictive eating patterns evident in ARFID are likely not driven by weight and shape concerns. Contrary to the hypothesis, the current study found no difference between AN and AAN on ED psychopathology, which is inconsistent with previous results that have demonstrated higher symptomatology among AAN compared to AN (e.g., Davenport et al. 2015; Fitterman‐Harris et al. 2024; Garber et al. 2019; Krug et al. 2022; Sawyer et al. 2016; Walsh et al. 2023; Zanna et al. 2021). Of note, the different findings to the current study have been found mostly in adult and/or inpatient settings, which may not generalise to adolescent outpatient settings, as Keery et al. (2019) found similar results to the current in an outpatient adolescent cohort. One explanation for this may be that, due to age differences, adolescent compared to adults have had less exposure to weight stigmatisation through their lives, which may lead to less internalised weight stigma and related weight and shape concerns among AAN. However, this hypothesis requires further exploration to confirm.

The findings suggest that there are no differences between subjective experience and severity of AN and AAN, at least as evaluated by the utilised measures. Combined with the lack of differences observed in comorbid symptomology, this provides more support for recent criticism of weight‐based distinctions between the diagnoses (e.g., Walsh et al. 2023). Although more data are needed to establish similarities and differences among these groups on the course, outcome, and response to treatments, at this stage, it appears warranted to consider removing the weight criterion separating the diagnosis, and instead looking toward other more empirically and clinically meaningful ways of distinguishing the AN spectrum disorders (if indeed distinction is required). For example, research has highlighted the utility of classifying EDs according to personality‐based tendencies toward overcontrol (i.e., obsessive, compulsive, perfectionistic) and under‐control (i.e., impulsive, mood intolerant; Dufresne et al. 2023), which have been shown to moderate treatment outcomes (Haynos et al. 2017). This may represent a more meaningful way to identify subgroups among EDs that would have significance for treatment allocation and may also serve to reduce the weight stigma induced by categorising according to weight (Harrop et al. 2023). Additionally, the complex and overlapping symptomology found in EDs may be well suited to being conceptualised according to dimensional models (i.e., Research Domain Criteria, Hierarchical Taxonomy of Psychopathology: Insel et al. 2010; Kotov et al. 2017) rather than the categorical approach of the DSM‐5. Such approaches may allow for more nuanced insights into potential mechanisms explaining the heterogeneity within EDs, which may further inform treatments.

### 6.3 Similarities and Difference on Age and BMI Centiles

Similar to inpatient settings, results showed that the ARFID group was younger than the AN and AAN groups, supportive of ARFID being a comparatively earlier onset disorder (Lieberman et al. 2019; Zanna et al. 2021). Additionally, the AAN group were of a higher BMI centile than the AN and ARFID groups, which was expected and consistent with previous results (Zanna et al. 2021). The ARFID and AN groups were not significantly different on BMI centile. These findings are consistent with previous results indicating no difference in BMI centiles between the ARFID and AN groups (e.g., Keery et al. 2019; Lieberman et al. 2019); however, they differ from studies that report lower BMI centiles for the AN group (e.g., Zanna et al. 2021). Importantly, the BMI results of the current study would be different if those with a greater than 15% weight loss for expected BMI centile were not included in the AN group and a ‘hard’ BMI centile of 5 was used. If this had been applied, the results would have replicated those of Zanna et al. (2021). When replicating the main analyses utilising this approach, the general interpretation of comorbid similarities and differences did not differ, which supports recent criticisms of weight cut‐offs to distinguish AN and AAN (Matthews et al. 2024).

### 6.4 Implications and Limitations

The current study was the first to compare AN, AAN and ARFID groups on a large range of comorbidity measures in an Australian outpatient sample. Taken together, these results have important implications for future research, especially related to treatments for AAN and ARFID, of which far less is known compared to AN. Given the similarities between AN and AAN, it may be that little or no diagnosis‐based adaptations are required to well established treatments for AN. Rather, it may be warranted to tailor treatment to the individual rather than the diagnosis based on existing recommendations (i.e., including additional emotional based supports for families with high expressed emotion in FBT; utilising the broad form for high perfectionism in CBT‐E). The current results also question the diagnostic utility of differentiating AN from AAN based on BMI. Additionally, for ARFID, it may be that comorbid symptomology may present less of a treatment barrier compared to AN and AAN and may require less or different accommodation or modification. Future research should investigate the role of comorbid symptomology in treatment outcome.

The current study has several limitations. Whilst ANOVA is relatively robust against uneven sample sizes, especially when homogeneity of variance is intact as in this study, our smaller ARFID group reduced power to detect group differences. Additionally, no specific self‐report questionnaires designed to capture ARFID were used, which limits the ability to capture ARFID specific symptomology. Furthermore, the sample was predominantly female, and there was no data collected on diversity of the sample (e.g., ethnicity, gender identity), potentially restricting the generalisability of the finding to diverse populations. Self‐report measures were used to assess comorbid symptomology, rather than standardised diagnostic interviews, which prevents accurate determination about the number of participants who met full diagnostic criteria. There was some attrition in completion of the measures due to the length of the battery, which is reflected in the variable sample sizes. There was no data collected on the degree to which the young people were malnourished (e.g., weight suppression, physical indicators of starvations), which prevents conclusions being drawn regarding associations with the comorbid symptoms reported. Future research could track symptomology across treatment and weight restoration. Lastly, no data on the duration of illness was collected, limiting the ability to test if comorbidity differences occur as a result of illness duration as opposed to diagnosis.

## 7 Conclusions

The current study was unique in examining similarities and differences between adolescents with AN, AAN or ARFID on the widest range of comorbid symptoms and psychological constructs to date. Findings from the study found little to no differences between AN and AAN aside from BMI centiles. Conversely, the current results clearly show a different and less severe comorbid symptom profile among those with ARFID. Overall, these findings provide a better understanding of the nature of comorbid symptomology among these disorders and highlight areas of future research that may provide further insight as to the role that they play in the treatment outcome.

## Author Contributions

D.W. conceptualised the study, analysed the data and wrote the original draft. T.W., N.L. supervised methodology and conceptualisation and were contributors to the writing, reviewing and editing of the manuscript. G.K., R.M., and M.D. were contributors the writing, reviewing and editing of the manuscript. All authors read and approved the final manuscript.

## Ethics Statement

The project was approved by the Children's Health Queensland Hospital and Health Service Human Research Ethics Committee (EC00175) HREC/20/QCHQ/67708. Informed consent was obtained from parents/legal guardians as per ethical approval.

## Consent

The authors have nothing to report.

## Conflicts of Interest

The authors declare no conflicts of interest.

## Data Availability

The datasets generated and/or analysed during the current study are not publicly available due to the conditions of ethical approval, but are available from the corresponding author on reasonable request.
